# Dropouts in randomized clinical trials of Korean medicine interventions: a systematic review and meta-analysis

**DOI:** 10.1186/s13063-021-05114-x

**Published:** 2021-03-01

**Authors:** Sae-rom Jeon, Dongwoo Nam, Tae-Hun Kim

**Affiliations:** 1 Department of Clinical Korean Medicine, Graduate School, Kyung Hee University, 26 Kyung Heedae-ro, Dongdaemun-gu, Seoul, 02447 Republic of Korea; 2 Department of Korea8n Medicine Clinical Trial Center, College of Korean Medicine, Kyung Hee University, 23 Kyung Heedae-ro, Dongdaemun-gu, Seoul, 02447 Republic of Korea

**Keywords:** Randomized clinical trials, Dropouts, Korean medicine, Acupuncture, Herbal medicine, Systematic review, Meta-analysis, Risk difference

## Abstract

**Background:**

The dropout rate is an important determinant of outcomes in randomized controlled trials (RCTs) and should be carefully controlled. This study explored the current dropout rate in studies of Korean medicine (KM) interventions by systematic evaluation of RCTs conducted in the past 10 years.

**Methods:**

Three clinical trial registries (Clinical Research Information Service, ClinicalTrials.gov, and World Health Organization International Clinical Trials Registry Platform) were searched to identify RCT protocols for KM interventions, such as acupuncture, herbal medicine, moxibustion, or cupping, and studies of mixed interventions, registered in Korea from 2009 to 2019. The PubMed, Embase, and OASIS databases were searched for the full reports of these RCTs, including published journal articles and theses. Dropout rates and the reasons for dropping out were analyzed in each report. The risk of bias in each of the included studies was assessed using the Cochrane risk of bias tool. The risk difference for dropping out between the treatment and control groups was calculated with the 95% confidence interval in a random effects model.

**Results:**

Forty-nine published studies were included in the review. The median dropout rate was 10% in the treatment group (interquartile range 6.7%, 17.0%) and 14% in the control group (interquartile range 5.4%, 16.3%) and was highest in acupuncture studies (12%), followed by herbal medicine (10%), moxibustion (8%), and cupping (7%). Loss to follow-up was the most common reason for dropping out. The risk difference for dropping out between the intervention and control groups was estimated to be 0.01 (95% confidence interval − 0.02, 0.03) in KM intervention studies.

**Conclusions:**

This review found no significant difference in the dropout rate between studies according to the type of KM intervention. We recommend allowance for a minimum dropout rate of 15% in future RCTs of KM interventions.

**Review protocol registration:**

PROSPERO CRD42020141011

**Supplementary Information:**

The online version contains supplementary material available at 10.1186/s13063-021-05114-x.

## Introduction

Dropout in the context of a clinical trial refers to a state in which observation is suspended or lost because a study participant cannot or does not attend the scheduled visits required by the research plan [1]. In most clinical trials, data are collected longitudinally. When data are collected repeatedly over a period of time, a proportion of the data will be lost if participants drop out of the study [2]. Dropping out is more common in subjects receiving interventions with potentially negative effects and might lead to incorrect estimation of the true effects of an intervention [3]. Loss of study data due to dropouts potentially introduces a risk of bias, so reducing the dropout rate is essential for a successful clinical trial. Furthermore, it is important to calculate an adequate sample size when designing a clinical trial in terms of research ethics while maintaining power and minimizing the exposure of patients to unnecessary risk [4, 5]. In terms of the quality, duration, and financial and ethical aspects of a clinical trial, understanding the relevant features of dropout is essential in the planning stages [6].

According to the *Yearbook of Traditional Korean Medicine*, updated in 2019, clinical research in the field of Korean medicine (KM) has been steadily increasing in the past two decades [7, 8]. Considering this growth, it is timely to consider improving the quality of clinical trials. In this context, it is important to understand the potential for dropouts and to be able to prepare a good management plan in future clinical studies of KM interventions. Previous analyses of factors related to dropouts in Korean clinical trials [6, 9] have only included data from institutional review board reports involving limited numbers of institutions and clinical trials. These studies were unable to provide general information on key dropout-related factors, estimates of overall dropout rates, or differences in dropout rates between various KM interventions.

The aims of this systematic literature review and meta-analysis were to identify factors affecting the likelihood of dropout in RCTs of KM interventions over a 10-year period and to estimate the potential dropout rate in such studies. It is hoped that this information could be used when planning future clinical research in the field of KM.

## Methods

### Inclusion criteria

#### Types of studies

Given that the Clinical Research Information Service (CRIS) was established in Korea in 2010, the plan was to include all RCTs of KM interventions that were conducted in Korea from 2009 to March 2019. No restrictions on the blinding methodology used were imposed at the time of selection. Non-randomized clinical trials, clinical trial protocols, and as yet unpublished studies were excluded.

#### Types of participants

Subjects who had been randomly assigned to a KM intervention in a clinical study conducted in Korea and then deviated from the study time points before the end of treatment or evaluation after screening were included. No specific restrictions were placed in terms of the type of disease for which patients were receiving KM or their symptoms.

#### Types of intervention

The KM interventions used in the RCTs included acupuncture, moxibustion, cupping and embedding of catgut, and administration of extracts, herbal medicine, chuna, electroacupuncture, pharmaco-acupuncture, and bee venom. No specific restrictions were imposed regarding the use of each intervention, number of treatments, or treatment methods. If a KM intervention was used in combination with conventional therapy, it was classified as a mixed intervention. Studies that included three or more interventions were excluded.

#### Comparison of interventions

Any type of comparative intervention was included with no specific limitations.

#### Outcome measures

There were no specific restrictions on the types of outcome variables used in individual studies or on the timing of evaluation.

#### Search strategy

The literature search was performed in two stages. In the first stage, all RCTs planned in Korea as of March 2019 were identified using the CRIS, ClinicalTrials.gov, and World Health Organization International Clinical Trials Registry Platform (WHO ICTRP) clinical trial registries. These RCTs had to be identified one by one because there is no established method for searching for KM interventions in these registries under the tag of country or RCT for each type of KM intervention (acupuncture, moxibustion, cupping, embedding of catgut, administration of extracts, herbal medicine, chuna, electro-acupuncture, pharmaco-acupuncture, or bee venom therapy). The search strategy used is outlined in Supplementary File 1. In the second stage, each study identified by the initial screening was searched for in the PubMed, Ovid Embase, and Oriental Medicine Advanced Searching Integrated System (OASIS) databases by title, author, and research registration number to check if it was published. No language restrictions were imposed. Relevant dissertations and articles published in journals from 2009 to March 2019 were included.

#### Study selection

One researcher (SRJ) conducted the literature search, and two researchers (THK and DWN) assessed the eligibility of the studies identified for inclusion in the analysis. The individual research results corresponding to the list extracted from the clinical research registries and the original text of the published papers were checked.

#### Data extraction and management

Two researchers (SRJ and DWN) extracted data from the included studies, and any disagreement was resolved by discussion. The data extracted from these studies included the first author, year of publication, type of institution, number of participating institutions, blinding methodology, and number of subjects in the treatment group and control group, as well as the total number of subjects, study design, type of intervention used in the KM group and control group, diseases or conditions treated, total number of treatments, frequency of assessment, and source of research funding. The reason for dropout was classified as withdrawal of consent, occurrence of an adverse event or serious adverse event, loss to follow-up, intervention discontinued, violation of eligibility criteria, protocol deviation, or others. The total number of dropouts from the treatment group and from the control group in each study was extracted.

#### Risk of bias assessment

Two reviewers (SRJ and DWN) independently assessed the risk of bias in each study using the Cochrane Handbook criteria. In the event of disagreement between the reviewers, the final decision was made by a third reviewer (THK). The risks of bias were evaluated in seven domains, including sequence generation, allocation concealment, blinding of research personnel, blinding of study participants, incomplete outcome data, selective reporting of outcomes, and other biases. The results of this evaluation were graded as “high risk of bias,” “low risk of bias,” or “unclear risk of bias” [10, 11].

### Data synthesis and meta-analysis

#### Primary analysis

The number and percentage of dropouts, reasons for dropout, and comparative dropout rates were analyzed according to the type of intervention in a narrative fashion. The proportion of overall dropouts to intervention-specific dropouts was calculated as the median and interquartile range (IQR) using the RStudio software (version 5.3.0; R Foundation for Statistical Computing, Vienna, Austria).

The risk difference (RD) was calculated to obtain a summary estimate of dropouts between the groups. The reason for using RD as a summary estimate was that the risk ratio or odds ratio was assumed to be unavailable because there could be a group without any dropouts in the included studies [11]. In this review, a random effects model was used for the meta-analysis because significant clinical heterogeneity between the individual studies had been expected due to considerable differences in the study design and performance. The meta-analysis was conducted using Review Manager (version 5.3.5 for Windows; The Nordic Cochrane Centre, Copenhagen, Denmark, 2014).

#### Subgroup analysis

A subgroup analysis was performed according to the intervention used (acupuncture, herbal medicine, moxibustion, cupping, and mixed). A sensitivity analysis was conducted according to whether the study was blinded or open and whether it was single-center or multicenter to identify the factors influencing the summary effect estimates.

#### Analysis of heterogeneity

Heterogeneity was evaluated using the chi-square test and the *I*^2^ statistic. In the chi-square test, a significance level of 0.10 was used. For the evaluation of the *I*^2^ statistic, the following criteria were used: 0% ≤ *I*^2^ ≤ 40%, “heterogeneity may not be important”; 30% ≤ *I*^2^ ≤ 60%, “may have moderate heterogeneity”; 50% ≤ *I*^2^ ≤ 90%, “may be actual heterogeneity”; and 75% ≤ *I*^2^ ≤ 100%, “significant heterogeneity” [10, 11].

#### Publication bias

We planned to assess a funnel plot visually to determine whether there was publication bias if more than 10 studies could be included in the meta-analysis [11].

## Results

**Fig. 1:**
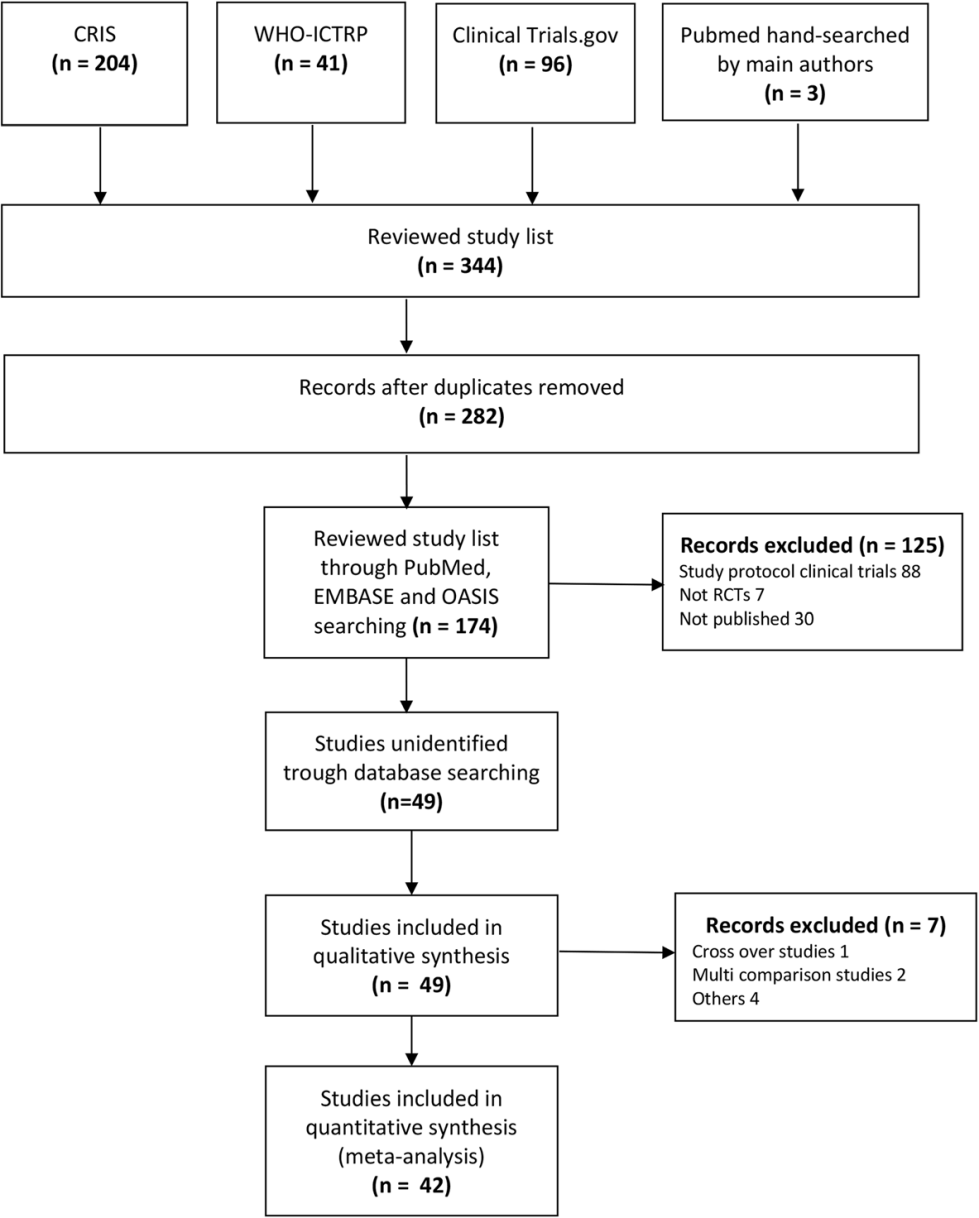
Flow chart showing the study selection process

The search of the clinical trial registries initially yielded 174 studies of interest. After screening, 49 studies (2943 participants) were eligible for inclusion in this review (Fig. 1) [12–60]. The relevant details of these studies are summarized in Table 1.

**Table 1:** Summary of the characteristics of the randomized controlled trials included in the meta-analysis

Author (year)	Protocol	Institutions, *n*	Blinding	Sample size (T/C)	Study design	Treatment intervention	Control intervention	Disease	Severity	Treatment period	Treatments, *n*	Funding
Choi (2010) [12]	Multicenter	4	Single	175 (116/59)	Parallel group	Acupuncture	Usual care	Perimenopause and post-menopause	NR	4 weeks	12	Yes
Choi (2012) [13]	Multicenter	3	Open	150 (75/75)	Parallel group	Acupuncture	Usual care	Dry eye	Other	8 weeks	12	Yes
Choi (2012) a [14]	Single-center	1	Single	42 (14/28)	Parallel group	Acupuncture	Distant and combination point	Temporomandibular joint disorder	Other	2 weeks	6	Yes
Choi (2015) [15]	Single-center	1	Single	30 (15/15)	Parallel group	Acupuncture	No treatment	Hypertension	Other	8 weeks	16	Yes
Choi (2015) a [16]	Multicenter	4	Open	150 (49/51)	Parallel group	Acupuncture	Sham acupuncture	Fatigue	Chronic	4 weeks	10	Yes
Choi (2018) [17]	Single-center	1	Single	30 (15/15)	Parallel group	Acupuncture	Sham acupuncture	Functional constipation	Other	4 weeks	12	Yes
Choi (2018) a [18]	Multicenter	4	Single	126 (63/63)	Parallel group	Acupuncture	Placebo	Diabetic peripheral neuropathy	Other	8 weeks	16	Yes
Han (2018) [19]	Single-center	1	Single	40 (20/20)	Parallel group	Embedding acupuncture	Acupuncture	Low back pain	Chronic	8 weeks	T4/C16	Yes
Jung (2009) [20]	Single-center	1	Double	52 (27/25)	Parallel group	Acupuncture	Sham acupuncture	Stroke	Other	3 days	1	Yes
Kim (2010) [21]	Single-center	1	Double	60 (30/30)	Parallel group	Pharmaco-acupuncture	Sham acupuncture	Osteoarthritis	Other	6 weeks	12	Yes
Kim (2018) b [22]	Multicenter	2	Open	16 (9/7)	Parallel group	Acupuncture	No treatment	Liver cirrhosis	Other	4 weeks	12	Yes
Lee (2011) [23]	Single-center	1	Single	54 (57/57)	Parallel group	Acupuncture	Sham acupuncture	Perimenopause and post-menopause	Other	7 weeks	11	Yes
Lee (2013) [24]	Single-center	1	Single	45 (15/30)	Parallel group	Acupuncture	Usual care	Neck pain	Chronic	3 weeks	9	Yes
Lee (2018) [25]	Single-center	1	Double	30 (15/15)	Parallel group	Acupuncture	Sham acupuncture	Cancer pain	Other	3 weeks	NR	Yes
Nam (2017) [26]	Single-center	1	Open	45 (13/29)	Parallel group	Acupuncture	Electro-acupuncture	Tinnitus	Other	4 weeks	8	No
Park (2014) [27]	Single-center	1	Single	61 (31/30)	Parallel group	Electro-acupuncture	Electro-acupuncture	Pain	Other	NR	1	Yes
Park (2016) [28]	Multicenter	2	Open	76 (37/39)	Other	Acupuncture	Acupuncture	Functional dyspepsia	Other	4 weeks	2	Yes
Shin (2018) [29]	Single-center	1	Single	39 (18/21)	Parallel group	Electro-acupuncture	Usual care	Low back pain	Chronic	4 weeks	8	Yes
Song (2013) [30]	Multicenter	3	Double	130 (65/65)	Parallel group	Acupuncture	Sham acupuncture	Low back pain	Chronic	6 weeks	12	Yes
Yang (2016) [31]	Single-center	1	Open	50 (26/24)	Parallel group	Acupuncture	Usual care	Lumbar spinal stenosis	Other	6 weeks	72–96	Yes
Yoo (2015) [32]	Single-center	1	Double	14 (7/7)	Parallel group	Acupuncture	Sham acupuncture	Thyroid cancer	Other	2 weeks	6	Yes
Chang (2018) [33]	Multicenter	2	Double	140 (70/70)	Parallel group	Herbal medicine	Placebo	Children with mild short stature	Other	24 weeks	336	N.R
Jung (2012) [34]	Single-center	1	Double	144 (73/71)	Parallel group	Herbal medicine	Placebo	Hwa-byung	Other	8 weeks	4	Yes
Kim (2011) [35]	Single-center	1	Double	166 (110/56)	Parallel group	Herbal medicine	Other	Obesity	Other	8 weeks	168	Yes
Kim (2013) [36]	Multicenter	2	Double	100 (50/50)	Parallel group	Herbal medicine	Placebo	Functional dyspepsia	Other	6 weeks	2	Yes
Kim (2014) [37]	Single-center	1	Double	50 (25/25)	Parallel group	Herbal medicine	Placebo	Obesity	Other	8 weeks	2 T/day	Yes
Kim (2016) [38]	Multicenter	2	Double	96 (48/48)	Parallel group	Herbal medicine	Placebo	Oral mucositis	Other	8 weeks	2	Yes
Kim (2019) [39]	Single-center	1	Double	60 (30/30)	Parallel group	Herbal medicine	Placebo	Allergic rhinitis	Other	4 weeks	2	Yes
Ko (2017) [40]	Single-center	1	Double	32 (16/16)	Parallel group	Herbal medicine	Placebo	Cancer-related anorexia	Other	4 weeks	2	Yes
Nah (2018) [41]	Multicenter	2	Double	143 (70/73)	Parallel group	Herbal medicine	Placebo	Knee osteoarthritis	Other	6 weeks	1	Yes
Son (2013) [42]	Single-center	1	Double	90 (60/30)	Parallel group	Herbal medicine	Placebo	Fatigue	Chronic	4 weeks	1	Yes
Yoon (2010) [43]	Single-center	1	Open	40 (20/20)	Parallel group	Herbal medicine	No treatment	Cancer-related fatigue	Other	2 weeks	1	Yes
Yoon (2018) [44]	Single-center	1	Open	30 (15/15)	Parallel group	Herbal medicine	No treatment	Cancer-related sleep disturbance	Other	2 weeks	1	Yes
Choi (2011) a [45]	Single-center	1	Single	26 (13/13)	Parallel group	Moxibustion	Sham moxibustion	Functional constipation	Other	4 weeks	12	Yes
Choi (2014) [46]	Multicenter	4	Open	212 (102/110)	Parallel group	Moxibustion	Usual care	Knee osteoarthritis	Other	4 weeks	4	Yes
Yoo (2017) [47]	Single-center	1	Single	16 (9/7)	Parallel group	Moxibustion	Sham moxibustion	Metastatic cancer	Other	2 weeks	10	Yes
Choi (2011) [48]	Single-center	1	Open	32 (21/11)	Parallel group	Cupping	No treatment	Low back pain	Other	2 week	6	Yes
Hong (2012) [49]	Single-center	1	Open	40 (20/20)	Parallel group	Cupping	Usual care	Neck pain	Other	2 weeks	6	Yes
Kim (2015) [50]	Single-center	1	Open	27 (15/12)	Parallel group	Other	Usual care	Hwa-byung	Other	4 weeks	4	Yes
Kim (2016) a [51]	Single-center	1	Single	14 (7/7)	Parallel group	Other	Placebo	Head and neck cancer	Other	8 weeks	4	Yes
Park (2018) [52]	Single-center	1	Double	73 (29/29)	Parallel group	Other	Sham	Parkinson’s disease	Other	12 weeks	24	Yes
Yoon (2016) [53]	Single-center	1	Single	30 (15/15)	Parallel group	Other	Usual care	Breast cancer	Other	Other	NR	Yes
Jung (2014) [54]	Multicenter	2	Double	147 (49/49/49)	Parallel group	Herbal medicine	Placebo	Anxiety disorder	Other	8 weeks	4	Yes
Kim (2018) [55]	Multicenter	2	Double	96 (32/32/32)	Other	Herbal medicine	Other	Functional dyspepsia	Other	8 weeks	1	Yes
Kim (2018) a [56]	Multicenter	2	Double	96 (64/32)	Parallel group	Herbal medicine	Placebo	Functional dyspepsia	Other	8 weeks	2	Yes
Kwon (2019) [57]	Single-center	1	Open	24 (12/12)	Crossover	Herbal medicine	–	Healthy male volunteers	Other	Other	1	NR
Park (2013) [58]	Single-center	1	Double	53 (13/14/14/12)	Other	Herbal medicine	Other	Diarrhea-dominant irritable bowel syndrome	Other	8 weeks	1	Yes
Kwon (2018) [59]	Single-center	1	Open	28 (14/14)	Crossover	Moxibustion	–	Overactive bladder	Other	4 weeks	8–12	NR
Kim (2012) [60]	Single-center	1	Open	44 (11/11/11/11)	Other	Other	Other	Acne vulgaris	Other	4 weeks	8	NR

*C* control group, *NR* not reported, *T* treatment group

Thirty-four of the 49 RCTs were performed at a single center [14, 15, 17, 19–21, 23–27, 29, 31, 32, 34, 35, 37, 39, 40, 42–45, 47–53, 57–60], and 15 were multicenter [12, 13, 16, 18, 22, 28, 30, 33, 36, 38, 41, 46, 54–56]. Twenty RCTs were performed in a double-blind manner [20, 21, 25, 30, 32–42, 52, 54–56, 58], and 14 were single-blind [12, 14, 15, 17–19, 23, 24, 27, 29, 45, 47, 51, 53]. Fifteen studies were performed without any blinding of participants or researchers [13, 16, 22, 26, 28, 31, 43, 44, 46, 48–50, 57, 59, 60]. Thirty-nine studies had a parallel design with a single control group [12–22, 25–27, 29–41, 43–51, 53, 55, 56], six included three or more parallel intervention groups [23, 24, 42, 52, 54, 58], and two had a crossover design [57, 59]. Acupuncture was the most frequently investigated KM and was assessed in 21 studies (*n* = 195) [12–32]. Herbal medicines were investigated in 17 studies (*n* = 132) [33–44, 54–58], moxibustion in four (*n* = 25) [45–47, 59], cupping therapies in two (*n* = 5) [48, 49], and mixed interventions in five (*n* = 12) [50–53, 60] (Supplementary File 2). Forty-four studies received funding support from the government, research institutions, or schools [12–25, 27–32, 34–56, 58]. Only one study was not supported by any external funding [16]. Four studies did not provide any information on funding [33, 57, 59, 60] (Supplementary File 3).

The treatment duration in the studies ranged from 2 to 24 weeks. The median treatment duration was 4 weeks after the exclusion of four studies that did not mention the treatment duration [20, 27, 53, 57].

Seven of the 49 studies eligible for inclusion in the review were excluded from the meta-analysis because no dropouts could be confirmed (*n* = 3), the study included more than three groups (*n* = 2), or it had a crossover design (*n* = 2). Data for the remaining 42 studies were included in the meta-analysis.

### Risk of bias in the included studies

A majority of the included studies showed a low risk of bias in most domains. Sequence generation was conducted appropriately using a random number table or by coin tossing, and allocation concealment was achieved adequately in the studies using sealed opaque envelopes. However, in the open trials, the risk of bias was evaluated as high or unclear in the domains concerning blinding of research personnel and participants [12, 13, 16, 22, 26, 28, 29, 31, 43, 44, 46, 48–50, 57]. Selective outcome reporting and other domains showed a low risk of bias in most studies (Supplementary Files 4 and 5).

### Analysis of dropouts

The total number of dropouts in all studies was 369, with the highest number in studies of acupuncture. There were 188 dropouts in the treatment groups, 80 (43%) of which were from studies of acupuncture, 74 (39%) were from herbal medicine studies, 25 (13%) were from studies of moxibustion, three (2%) were from cupping studies, and six (3%) were from mixed interventions. Of the 181 dropouts from the control groups, 102 (56%) were from acupuncture studies, 58 (32%) were from herbal medicine studies, 13 (7%) were from moxibustion studies, two (1%) were from cupping studies, and six (3%) were from mixed intervention studies.

The reported overall dropout rate was 10% (IQR 6.7%, 17.0%) in the treatment group, 12% (IQR 7.9%, 16.5%) in the control group, and 12% (IQR 7.9%, 16.5%) in all groups. When classified by type of intervention, the median dropout rate was 12% (IQR 10.8%, 21.3%) for acupuncture studies, 10% (IQR 7.2%, 14.0%) for herbal medicine studies, 8% (IQR 8.1%, 19.9%) for moxibustion studies, 7% (IQR 6.1%, 8.3%) for cupping studies, and 4% (IQR 2.5%, 7.1%) for mixed intervention studies (Table 2).

**Table 2:** Dropout rate according to the type of Korean medicine intervention

Study category (number)	Treatment group: overall and median dropout rates (IQR)	Control group: overall and median dropout rates (IQR)	Total: overall and median dropout rates (IQR)
Acupuncture (*n* = 21)	$\frac{93}{687}$, 14% (6.9%, 22.2%)	$\frac{102}{710}$, 14% (9.9%, 18.6%)	$\frac{195}{1397}$, 12% (10.8%, 21.3%)
Herbal medicine (*n* = 12)	$\frac{74}{587}$, 10% (7.8%, 13.0%)	$\frac{58}{504}$, 10% (5.7%, 15.0%)	$\frac{132}{1091}$, 10% (7.2%, 14.0%)
Moxibustion (*n* = 3)	$\frac{12}{124}$, 15% (10.1%, 35.5%)	$\frac{13}{130}$, 0% (0.0%, 5.9%)	$\frac{25}{254}$, 8% (8.1%, 19.9%)
Cupping (*n* = 2)	$\frac{3}{41}$, 7% (6.1%, 8.4%)	$\frac{2}{31}$, 7% (6.0%, 8.1%)	$\frac{5}{72}$, 7% (6.1%, 8.3%)
Mixed intervention (*n* = 4)	$\frac{6}{66}$, 3% (0.0%, 9.3%)	$\frac{6}{63}$, 3% (0.0%, 9.3%)	$\frac{12}{129}$, 4% (2.5%, 7.1%)
Total (*n* = 42)	$\frac{188}{1505}$, 10% (6.7%, 17.0%)	$\frac{181}{1438}$, 14% (5.4%, 16.3%)	$\frac{369}{2943}$, 12% (7.9%, 16.5%)

*IQR* interquartile range

Loss to follow-up was the most common reason for dropouts overall. When examined by type of intervention, loss to follow-up was the most common reason for dropout in studies using acupuncture, herbal medicine, and cupping whereas withdrawal of consent was the most frequent reason in the moxibustion and other studies (Supplementary Files 6, 7, 8, 9, and 10).

### Meta-analysis on the dropout rates

**Fig. 2:**
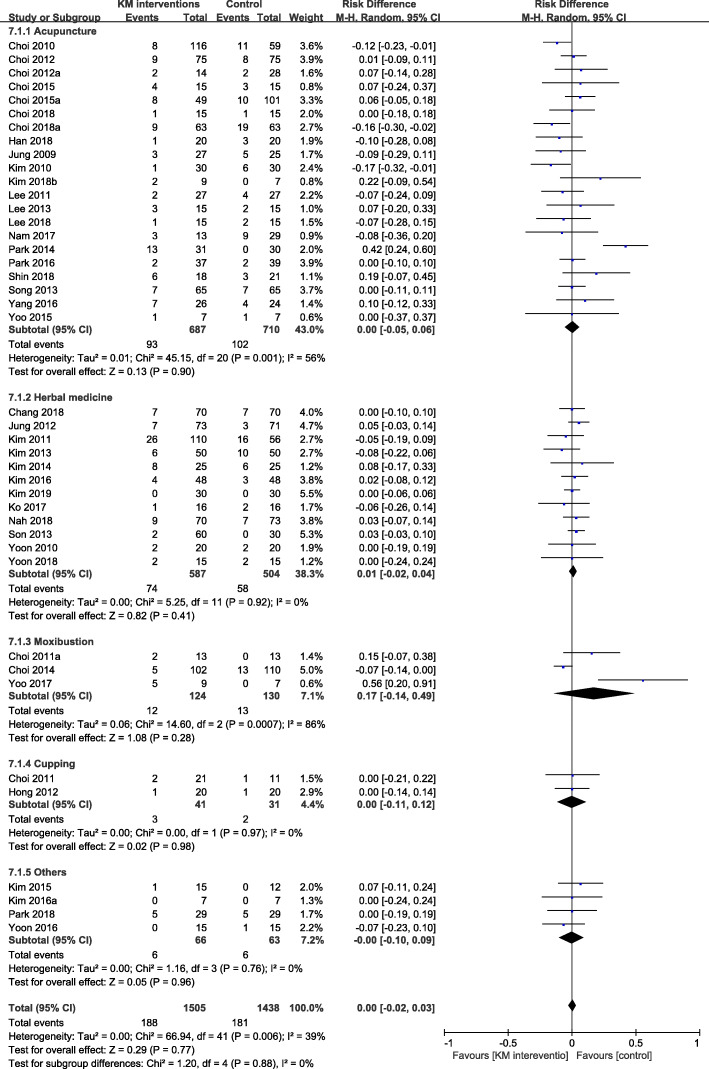
Forest plot comparing Korean medicine interventions and control interventions

The RD in dropout rates between the intervention and control groups was estimated to be 0.01 (95% confidence interval [CI] − 0.02, 0.03) in the 42 studies regardless of the type of intervention, suggesting no significant difference in the dropout rate. Moderate heterogeneity (*I*^2^ = 39%) was observed in the meta-analysis of studies of different interventions. The RD was estimated to be less than 0.01 (95% CI − 0.05, 0.06) in the acupuncture studies, 0.01 (95% CI − 0.02, 0.04) in the herbal medicine studies, 0.17 (95% CI − 0.14, 0.49) in the moxibustion studies, less than 0.01 (95% CI − 0.11, 0.12) in the cupping studies, and less than 0.01 (95% CI − 0.10, 0.09) in the studies with mixed interventions (Fig. 2).

### Subgroup analysis and publication bias

There was no significant difference in the RD for dropout according to whether the study design was blinded or open, whether it was single-center or multicenter, or whether or not the number of treatments administered was more than eight (the median number of visits in the included studies) (Table 3). Visual inspection of the funnel plot indicated no significant publication bias in the meta-analysis (Supplementary File 11).

**Table 3:** Subgroup analysis

Confounding factor	Subset	Summary effect estimate
Blinding	Blinded	RD 0.00, 95% CI (− 0.04 to 0.04)
	Open	RD 0.00, 95% CI (− 0.04 to 0.04)
Institution	Multicenter	RD − 0.02, 95% CI (− 0.06 to 0.02)
	Single-center	RD 0.02, 95% CI (− 0.02 to 0.06)
Duration of treatment	Less than 8 weeks	RD 0.01, 95% CI (− 0.03 to 0.05)
	More than 8 weeks	RD − 0.00, 95% CI (− 0.05 to 0.04)

*CI* confidence interval, *RD* risk difference

## Discussion

This systematic review investigated the status of dropouts from 49 RCTs of KM interventions between 2009 and 2019. The most common intervention was acupuncture (21 studies), followed by herbal medicine (17 studies), mixed interventions (five studies), moxibustion (four studies), and cupping (two studies). The estimated median dropout rate was 12% (IQR 7.9%, 16.5%) across the treatment and control groups. The most common reason for dropping out was loss to follow-up in the studies of acupuncture, herbal medicine, and cupping and withdrawal of consent in the moxibustion and other studies. A meta-analysis of all studies found no statistically significant RD in the dropout rate between the treatment and control groups; this finding remained the same when the data were analyzed by type of intervention, methodology (whether the study was blinded or not), and setting (single-center or multicenter).

The dropout rates identified in this review are slightly lower than those previously reported in the literature [8, 61, 62]. Moreover, the main reason cited for dropping out in a previous review was non-compliance with treatment [9], whereas we found loss to follow-up and withdrawal of consent to be the most common reasons. This inconsistency may reflect differences in the interventions used in the studies included in the different reviews. In a previous report, only studies using acupuncture were evaluated, whereas our review included various interventions.

This research has several strengths. First, it is the only systematic review and meta-analysis of dropouts from RCTs in the KM field. The reasons for dropping out were classified by the type of KM intervention, and the median dropout rates were estimated accordingly. A previous systematic review of studies that investigated structural outcomes in patients with rheumatic diseases found a dropout rate of more than 20%, which raises doubt regarding the validity of its findings [63]. Reasonable data for assuming a potential dropout rate are critical when calculating the sample size. Second, our systematic review included studies performed at different institutions in Korea; therefore, our findings could be generalized to all of Korea. It would be impossible to determine the overall status of dropouts from RCTs by simply analyzing research documents at specific institutions. However, this study differs from the previous studies in that the reasons for dropouts in the individual studies were classified and compared with the ratio of dropouts by interventions and the risk of dropouts between the treatment group and the control group through a systematic evaluation of previously published literature. Therefore, our present findings would be helpful when estimating the likely dropout rate for each type of KM intervention in future clinical studies.

There are also some limitations to this review. First, it analyzed secondary data extracted from previously published reports and did not include unpublished studies (i.e., gray literature). Second, many studies did not provide clear reasons for dropouts, which were categorized as unknown in this study. In these studies, the exact reason for the dropouts could not be confirmed, which precluded the drawing of a definite conclusion. Third, this research was preliminary in nature and the only such study ever conducted in Korea, so may not reflect the real-world situation in other countries, where the findings for other types of KM intervention may be different. Fourth, the analysis according to the type of intervention might have been affected by the number of included studies. For example, our finding that RCTs of acupuncture had the highest dropout rates may simply reflect the fact that our study included a large number of acupuncture studies. Moreover, there could have been factors or predictors related to the dropouts in the KM intervention trials that we could not identify. This possibility should be evaluated in the future.

Several findings of this research warrant further discussion. First, adverse events were not found to be a common reason for dropping out of the KM intervention trials. The dropout rate due to adverse events was found to be 5% in this study. Second, the type of intervention used in control subjects might not be an important determinant of the risk of dropping out. Non-compliance is widely thought to indicate dissatisfaction with treatment. However, in the studies that included a sham control group, the median dropout rate was estimated to be 11% (IQR 9.3%, 15.4%) across the treatment and control groups and was not significantly different from that in studies that used other types of control interventions (the estimated median dropout rate for all studies included in this review was 12% [IQR 7.9%, 16.5%]). When planning an RCT, investigators should consider specific design factors likely to affect the dropout rate, including frequency of visits, follow-up assessments, and type of intervention. The inclusion of a sham control group might not be an important factor in terms of an increased dropout rate. Finally, only 49 of the 174 potentially eligible KM studies entered into the clinical trial registries in the past 10 years have been published, suggesting a publication rate of about 28%. However, our search strategy may not have identified the exact number of relevant studies, which might have introduced a degree of bias. Nevertheless, it is clear that a substantial amount of research in the field of KM have not been formally published.

In this research, we examined methodological factors that might increase the dropout rate, such as blinding, whether or not the study was single-center or multicenter, and treatment frequency, but could not identify any such factors. It is uncertain whether this negative result reflects a lack of studies; moreover, it is unclear whether they are actually relevant. Dropout-related factors should be examined in a more extensive review that includes a larger number of clinical studies in the future or alternatively by surveys of actual dropouts in the clinical trials presently underway.

## Conclusions

This review and meta-analysis of RCTs in which KM interventions were used revealed a dropout rate of less than 15% over a 10-year period and found no statistically significant difference in the dropout rate between the treatment and control groups. These data can be used to calculate the likely dropout rate when designing a clinical trial using KM. Further studies are needed to develop a strategy for reducing the factors affecting dropout rates.

## Supplementary Information

**Additional file 1.** : Search strategy used for the Clinical Research Information Service, ClinicalTrials.gov, and World Health Organization International Clinical Trials Registry Platform registries.

**Additional file 2.** : Number of included studies listed by type of intervention.

**Additional file 3.** : Summary of the included randomized controlled trials.

**Additional file 4.** : Risk of bias assessment.

**Additional file 5.** : Risk of bias assessment summary for the individual studies.

**Additional file 6.** : Reasons for dropping out in the 21 studies of acupuncture.

**Additional file 7.** : Reasons for dropping out in the 12 studies of herbal medicine.

**Additional file 8.** : Reasons for dropping out in the three studies of moxibustion.

**Additional file 9.** : Reasons for dropping out in the two studies of cupping.

**Additional file 10.** : Reasons for dropping out in the four studies of mixed interventions.

**Additional file 11.** : Funnel plot assessing publication bias.

## Data Availability

All data and materials supporting the conclusions of this research are included in the article.

## Abbreviations

KM: Korean medicine
RCT: Randomized controlled trial
CRIS: Clinical Research Information Service
WHO ICTRP: World Health Organization International Clinical Trials Registry Platform
ROB: Risk of bias
RD: Risk difference
OASIS: Oriental Medicine Advanced Searching Integrated System
IQR: Interquartile range
CI: Confidence interval
AE: Adverse event
SAE: Serious adverse event
