# Supplementary material for: Dropouts in randomized clinical trials of Korean medicine interventions: a systematic review and meta-analysis
Source: Trials. 2021 Mar 1;22:176. doi: 10.1186/s13063-021-05114-x (PMC7923634; doi:10.1186/s13063-021-05114-x)
Supplement: Supplementary file 1 — Additional file 1. : Search strategy used for the Clinical Research Information Service, ClinicalTrials.gov, and World Health Organization International Clinical Trials Registry Platform registries. [file 13063_2021_5114_MOESM1_ESM.docx]

Supplementary File 1. Search strategy used for the Clinical Research Information Service, ClinicalTrials.gov, and World Health Organization International Clinical Trials Registry Platform registries.

Search strategy of the CRIS (2009 – February 2019)

Search Strategy:

1 Research protocols containing a keyword, "random" in titles regarding Korean Medicine were searched.

2 Research protocols will be searched individually with the following terms (#1~11)

#1 Acupuncture

#2 Chuna

#3 Embedding

#4 Electroacupuncture

#5 Moxibustion

#6 Pharmacoacupuncture

#7 Cupping

#8 Korean Medicine

#9 Herbal

#10 Bee venom

#11 Extract

3 The research protocols identified through searching will be included if it is conducted with randomization methods.

Search strategy of the ClinicalTrials.gov (2009 – February 2019)

Search Strategy:

1 Country Korea, Republic of AND (Random or RCT) AND #1~11 (Individually)

2 Country Korea, Republic of AND #1~11 (Individually, research protocols were included if it is conducted with randomization process)

#1 Acupuncture

#2 Chuna

#3 Embedding

#4 Electroacupuncture

#5 Moxibustion

#6 Pharmacoacupuncture

#7 Cupping

#8 Korean Medicine

#9 Herbal

#10 Bee venom

#11 Extract

Search strategies of the WHO-ICTRP (2009 – February 2019)

Search Strategy:

1 Countries of recruitment are Republic of Korea AND #1~11 (Individually)

2 Countries of recruitment are Republic of Korea AND #1~11 (Individually) AND (Random OR RCT)

#1 Acupuncture

#2 Chuna

#3 Embedding

#4 Electroacupuncture

#5 Moxibustion

#6 Pharmacoacupuncture

#7 Cupping

#8 Korean Medicine

#9 Herbal

#10 Bee venom

#11 Extract
