# Supplementary figures and images for: Dropouts in randomized clinical trials of Korean medicine interventions: a systematic review and meta-analysis
Source: Trials. 2021 Mar 1;22:176. doi: 10.1186/s13063-021-05114-x (PMC7923634; doi:10.1186/s13063-021-05114-x)

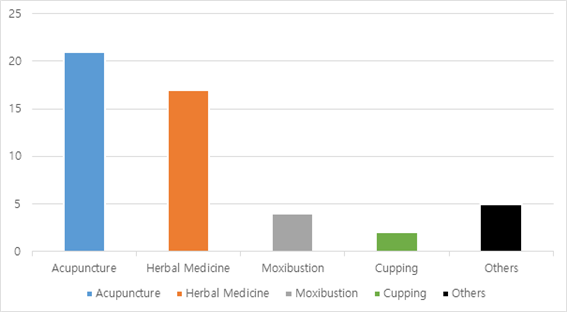

Supplement: Supplementary file 2 — Additional file 2. : Number of included studies listed by type of intervention. [file 13063_2021_5114_MOESM2_ESM.tiff]

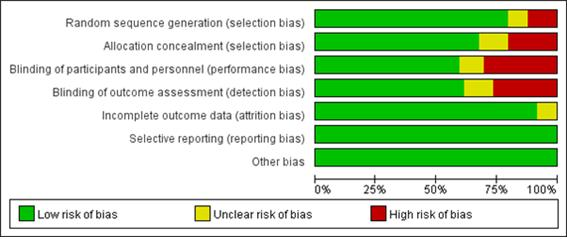

Supplement: Supplementary file 4 — Additional file 4. : Risk of bias assessment. [file 13063_2021_5114_MOESM4_ESM.tif]

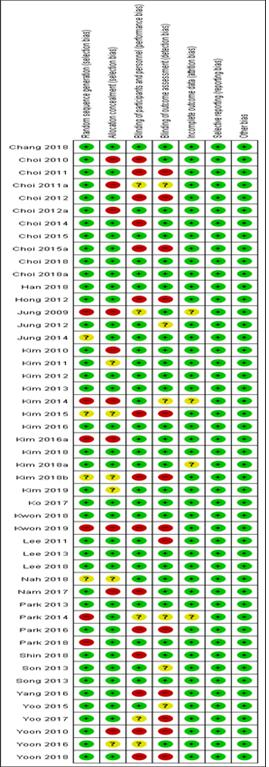

Supplement: Supplementary file 5 — Additional file 5. : Risk of bias assessment summary for the individual studies. [file 13063_2021_5114_MOESM5_ESM.tif]

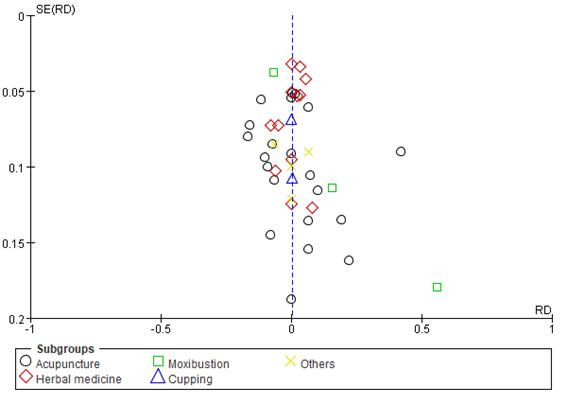

Supplement: Supplementary file 11 — Additional file 11. : Funnel plot assessing publication bias. [file 13063_2021_5114_MOESM11_ESM.tiff]
