# Supplementary material for: Dropouts in randomized clinical trials of Korean medicine interventions: a systematic review and meta-analysis
Source: Trials. 2021 Mar 1;22:176. doi: 10.1186/s13063-021-05114-x (PMC7923634; doi:10.1186/s13063-021-05114-x)
Supplement: Supplementary file 3 — Additional file 3. : Summary of the included randomized controlled trials. [file 13063_2021_5114_MOESM3_ESM.docx]

Supplementary File 3. Summary of the included randomized controlled trials.

RCT : Randomized Controlled Trial

| **Year** | 2009-2011 | 8 |
| --- | --- | --- |
|  | 2012-2015 | 18 |
|  | 2016-2019 | 23 |
| **Institution** | Single-center | 34 |
|  | Multi-center | 15 |
| **Blind** | Single | 14 |
|  | Double | 20 |
|  | Open | 15 |
| **Study Design** | Two group parallel design RCT studies | 39 |
|  | More than three group parallel design RCT studies | 6 |
|  | Cross over studies | 2 |
|  | Other studies | 2 |
| **Intervention** | Acupuncture | 21 |
|  | Herbal Medicine | 17 |
|  | Moxibustion | 4 |
|  | Cupping | 2 |
|  | Other | 5 |
| **Stage** | Acute stage | 0 |
|  | Chronic stage | 6 |
|  | Unclear | 43 |
| **Funding Sources** | Yes | 44 |
|  | No | 5 |
