# Supplementary material for: Dropouts in randomized clinical trials of Korean medicine interventions: a systematic review and meta-analysis
Source: Trials. 2021 Mar 1;22:176. doi: 10.1186/s13063-021-05114-x (PMC7923634; doi:10.1186/s13063-021-05114-x)
Supplement: Supplementary file 6 — Additional file 6. : Reasons for dropping out in the 21 studies of acupuncture. [file 13063_2021_5114_MOESM6_ESM.docx]

Supplementary File 8. Reasons for dropping out in the 21 studies of acupuncture.

AE : Adverse Events / SAE : Severe Adverse Events

Protocol Deviation : treatment non-compliance, other combination treatments and drug violation are included

| **Reasons for the Drop-outs** | **Treatment Group** | **Control Group** |
| --- | --- | --- |
| Withdrawal of Consent | 16 | 23 |
| AE | 3 | 2 |
| SAE | 1 | 0 |
| Lost to Follow-up | 30 | 44 |
| Discontinued Intervention | 1 | 0 |
| Violation of Inclusion and Exclusion Criteria | 6 | 3 |
| Protocol Deviation | 18 | 7 |
| Other | 18 | 23 |
| **Total** | 93 | 102 |

Intradermal Acupuncture, Electro Acupuncture, Pharmaco Acupuncture, Embedding are included.
