# Supplementary material for: Dropouts in randomized clinical trials of Korean medicine interventions: a systematic review and meta-analysis
Source: Trials. 2021 Mar 1;22:176. doi: 10.1186/s13063-021-05114-x (PMC7923634; doi:10.1186/s13063-021-05114-x)
Supplement: Supplementary file 7 — Additional file 7. : Reasons for dropping out in the 12 studies of herbal medicine. [file 13063_2021_5114_MOESM7_ESM.docx]

Supplementary File 9. Reasons for dropping out in the 12 studies of herbal medicine.

AE : Adverse Events / SAE : Severe Adverse Events

Protocol Deviation : treatment non-compliance, other combination treatments and drug violation are included

| **Reasons for the Drop-outs** | **Treatment Group** | **Control Group** |
| --- | --- | --- |
| Withdrawal of Consent | 5 | 7 |
| AE | 8 | 4 |
| SAE | 2 | 0 |
| Lost to Follow-up | 27 | 19 |
| Discontinued Intervention | 6 | 10 |
| Violation of Inclusion and Exclusion Criteria | 0 | 0 |
| Protocol Deviation | 12 | 10 |
| Other | 14 | 8 |
| **Total** | 74 | 58 |

*Three studies which did not suggest drop-outs of treatment and control groups separately were excluded.

*One study which included more than four groups and 1 study which was a cross-over study were excluded (total 2 studies).
