# Supplementary material for: Dropouts in randomized clinical trials of Korean medicine interventions: a systematic review and meta-analysis
Source: Trials. 2021 Mar 1;22:176. doi: 10.1186/s13063-021-05114-x (PMC7923634; doi:10.1186/s13063-021-05114-x)
Supplement: Supplementary file 9 — Additional file 9. : Reasons for dropping out in the two studies of cupping. [file 13063_2021_5114_MOESM9_ESM.docx]

Supplementary File 11. Reasons for dropping out in the two studies of cupping.

AE : Adverse Events / SAE : Severe Adverse Events

Protocol Deviation : treatment non-compliance, other combination treatments and drug violation are included

| **Reasons for the Drop-outs** | **Treatment Group** | **Control Group** |
| --- | --- | --- |
| Withdrawal of Consent | 0 | 1 |
| AE | 0 | 0 |
| SAE | 0 | 0 |
| Lost to Follow-up | 3 | 1 |
| Discontinued Intervention | 0 | 0 |
| Violation of Inclusion and Exclusion Criteria | 0 | 0 |
| Protocol Deviation | 0 | 0 |
| Other | 0 | 0 |
| **Total** | 3 | 2 |
